# Who do you think you are? - Personality in eating disordered patients

**DOI:** 10.1186/s40337-015-0042-6

**Published:** 2015-03-11

**Authors:** Johanna Levallius, David Clinton, Martin Bäckström, Claes Norring

**Affiliations:** Resource Center for Eating Disorders, Department of Clinical Neuroscience, Karolinska Institutet, Norra Stationsgatan 69, 113 64 Stockholm, Sweden; Department of Psychology, Lund University, 221 00 Lund, Sweden; Stockholm Center for Eating Disorders, Research & DevelopmentCentre for Psychiatric Research, Karolinska institutet & Stockholm County Council, Stockholm, Sweden

**Keywords:** Five-factor model, NEO PI-R, Eating disorder, Bulimia nervosa, Eating disorder not otherwise specified

## Abstract

**Background:**

The Five-Factor Model of personality is strongly linked to common mental disorders. Yet the relationship between the lower order personality traits (facets) of the model and eating disorder (ED) features remains unclear. The aim of the study was to explore how patients with non-anorexic ED differ from controls in personality and to examine the ability of personality facets to explain psychopathology.

**Methods:**

Female patients with non-anorexic ED (N = 208) were assessed on general psychopathology, ED symptoms and personality as measured by the NEO PI-R; and were compared on personality to age-matched female controls (N = 94).

**Results:**

Compared to controls, patients were characterised by experiencing pervasive negative affectivity and vulnerability, with little in the way of positive emotions such as joy, warmth and love. Patients were also significantly less warm and sociable, and exhibited less trust, competence, and self-discipline. Finally, they were less open to feelings, ideas and new experiences, yet more open in their values. Among patients, personality facets explained up to 25% of the variance in ED and general psychopathology.

**Conclusions:**

ED patients have distinct patterns of personality. Identifying and focusing on personality traits may aid in understanding ED, help therapists enhance the treatment alliance, address underlying problems, and improve outcome.

## Background

Common mental disorders are strongly linked to personality and demonstrate similar trait profiles to one another [1]. In eating disorders (ED), personality has been shown to play a major role as a risk factor [2], as a moderator of symptomatic expression [3], for choice of treatment [4], and as a predictor of outcome [5-7]. Personality traits commonly associated with eating disorder (ED) are high perfectionism, impulsivity, harm avoidance, reward dependence, sensation seeking, neuroticism, and obsessive-compulsiveness in combination with low self-directedness, assertiveness, and cooperativeness [8-11]. Some traits are common regardless of particular eating disorder (ED), while others are more strongly related to certain types of ED, e.g. high perfectionism in anorexia nervosa (AN) and elevated sensation-seeking in patients who binge eat [12]. Some of the most salient risk factors for ED that have been identified are neuroticism and perfectionism [2], and there is also evidence that maladaptive personality traits of borderline, histrionic and schizotypal nature precede ED development [13]. Personality matters for outcome as well, for instance under-controlled/impulsive [7], and avoidant/insecure [14] patients have a poorer prognosis while high-functioning patients fare better than average [14]. Lastly, Fairburn and colleagues [4] have demonstrated that outcome is improved when addressing personality features in conjunction with ED symptoms. Even as ED patients recover, they appear to retain a personality pattern differing from that of women who have never had such disorders [6,9]. Unfortunately, the majority of previous work on personality in ED has utilized the Temperament and Character Inventory (TCI) or later revisions, which suffer from serious theoretical and psychometric shortcomings. Cloninger’s compelling theory of temperament having a high degree of genetic heritability whilst character dimensions being mainly under the influence of environmental pressures lacks empirical evidence. Further, the association of facets to specific domains has in several instances not been supported [15].

Few studies have assessed personality in ED patients based on the most validated and widely accepted personality taxonomy, i.e. the Five-Factor Model (FFM) [16]. The FFM has been shown to predict important life outcomes such as happiness, health, work satisfaction, job performance, quality of close relationships, and identity formation [17]. It also underlies the alternative model for personality disorders in the Diagnostic and Statistical Manual of Mental Disorders 5^th^ Edition (DSM-5). The FFM defines personality on five broad dimensions: Neuroticism, Extraversion, Agreeableness, Conscientiousness, and Openness to Experience. Each dimension, in turn, comprises six lower order personality traits, named facets [16]. From young adulthood and onward we are expected to mature in personality by gradually becoming more emotionally stable, conscientious, agreeable, and assertive, yet less gregarious and in a curvilinear fashion more and then less open to new experiences [18,19]. On the individual level, valid assessment of personality on the facet level is of important clinical value. Capturing a patients’ unique FFM profile provides a comprehensive description of an individual’s adaptive and maladaptive traits, making it useful in communication with patients and their family, as well as a valuable tool for treatment planning and in forecasting challenges to the treatment process [20].

The limited number of studies investigating FFM dimensions in ED patients have found a pattern of high Neuroticism, low Extraversion, low Agreeableness, and low Conscientiousness e.g. [3,21,22]. De Bolle and colleagues [21] compared in-patients with AN or bulimia nervosa (BN) on the thirty facets of the FFM. The only significant differences between them were that BN patients scored higher on Impulsiveness and lower on Deliberation, as one might expect. However, the utility of the study is limited by its’ focus on inpatients, encompassing a relatively small portion of the ED population. This study is to date the only published study of the complete FFM in ED patients. This is unfortunate since the literature on personality and psychopathology has repeatedly concluded that exploration at the dimensional level, instead of the facet level, is too crude, meaning that important information is lost [17,23]. As an example, Bienvenu and colleagues [24] discovered by facet-level analysis, that patients with social phobia were not low in the Agreeableness dimension in general but in the facet Trust specifically, and depressed patients were within normal range in Openness to Experience, though high on Openness to Feelings; vital knowledge in treatment. Given the lack of knowledge concerning the FFM in ED in general, and in particular the total lack of studies at the underlying facet level on outpatients, the present study aimed to explore how outpatients with non-anorexic ED differ from controls in personality facets as measured by the NEO PI-R and to examine the ability of personality facets to explain variance in ED and general psychopathology.

## Methods

### Participants

Patients with non-anorexic ED who were treated at the Stockholm Centre for Eating Disorders (SCED) between 2010 and 2013 were included in the study. SCED is Scandinavia’s largest ED treatment facility, comprising in-, day- and out-patient units, as well as a family unit and an acute mobile team. Approximately 700 patients of all ages are admitted each year. Potential participants in the present study had either been enrolled in a randomized control trial of internet-based CBT (N = 150) or enrolled in a multimodal day-patient treatment (N = 129). Total response rate was 76% (N = 102 and 109). All were adult females with DSM-IV diagnosis of Bulimia Nervosa or ‘Eating Disorder Not Otherwise Specified’ (EDNOS). Two patients failed to complete the personality inventory and one patient had Anorexia Nervosa and was therefore excluded, which left a total patient sample of N = 208 (BN N = 124, EDNOS N = 84). The patient group could be considered severe, since mean score on ED symptom questionnaire (EDE) corresponded to the 95^th^ percentile in young adult women [25]. Minimum body mass index for inclusion was 17.5. Mean age was 29.2 years (*SD* = 8.2). All patients provided an informed consent.

The control sample comprised female volunteers (N = 94); half were university students in various courses and programmes, and the other half were in low to highly qualified employment. Data were collected in 2007 and 2011 in a validity study of another inventory; participants were not screened for ED. Mean age was 28.8 years (*SD* = 9.2). The main purpose for contrasting personality with a control sample was to rule out age as a confounding factor.

### Measures

#### Eating Disorder Examination Questionnaire (EDE-Q)

Derived from the Eating Disorder Examination (EDE) interview, the EDE-Q provides a brief and comprehensive assessment of core eating disorders psychopathology [26]. It contains attitudinal items that form four subscales (Restraint, Eating Concern, Shape Concern, and Weight Concern), as well as items to measure frequency of laxative misuse, vomiting, excessive exercise and incidents of binge eating over the previous 28 days. The EDE has satisfactory psychometric properties [27].

#### Comprehensive Psychiatric Rating Scale - Self-rating Scale for Affective Syndromes (CPRS)

The CPRS consists of 19 items measuring anxiety, depression and compulsiveness [28], common features in ED patients. The instrument has demonstrated good psychometric properties [28,29].

#### Clinical Impairment Assessment (CIA; version 3.0)

The CIA is a 16-item questionnaire assessing the severity of psychosocial impairment in relation to ED behaviours and attitudes covering mood, self-perception, cognitive functioning, interpersonal functioning, and work performance during the last 28 days [30]. The CIA has good psychometric properties in clinical samples [30,31].

#### Structured Eating Disorder Interview (SEDI)

The SEDI is a semi-structured clinical interview developed specifically for detailed ED diagnosis. It is based directly on the DSM-IV ED criteria and consists of 20–30 questions, depending on what follow-up questions need to be asked due to presenting symptomatology [32]. Validation against the EDE-interview has shown a good concordance [32].

#### NEO Personality Inventory Revised, NEO PI-R

The NEO PI-R is a 240-item self-report measure designed to measure both the five dimensions and 30 facets of the FFM [16]. Respondents rate statements of behaviour, feelings and attitudes on a five point Likert scale from strongly disagree to strongly agree. The factor structure has been reproduced in a psychiatric sample [33], and the Swedish version shows satisfactory psychometric properties, with the exception of facet Openness to Values [34]. Cronbach’s alpha of facets in the control sample and the patient sample was on par with American norms, averaging α = .68 (range .40 to .90) and α = .73 (range .42 to .86) respectively. Coefficient alpha was below .60 in the same three facets of both samples namely Excitement-Seeking, Openness to Values, and Tender Mindedness. For overview and definition of facets see Table 1.

**Table 1:** **Overview of dimensions and facets of the five-factor model**

**Dimensions**	**Facet scales**	**Label**	**Definition**
Neuroticism	Anxiety	N1	Proneness to worry and rumination.
	Angry Hostility	N2	The readiness to experience frustration, anger and bitterness.
	Depression	N3	The tendency for guilt, sadness, loneliness and hopelessness.
	Self-Consciousness	N4	Sensitivity in social situations, such as ridicule, rejection or awkwardness.
	Impulsiveness	N5	The ability to tolerate frustration and to control urges, cravings, and desires.
	Vulnerability	N6	The ability to cope with stress.
Extraversion	Warmth	E1	The degree of displayed affection and closeness in relationships.
	Gregariousness	E2	The tendency to seek the company of others.
	Assertiveness	E3	The degree of dominance in social interaction.
	Activity	E4	The level of energy and activity in daily life.
	Excitement-Seeking	E5	The need for thrills and intense stimulation.
	Positive Emotions	E6	The tendency to be happy, excited and cheerful.
Openness to experience	Fantasy	O1	Proneness to imagination, day-dreaming, and creating.
	Aesthetics	O2	Appreciation for beauty in e.g. art, music, poetry or nature.
	Feelings	O3	Receptivity to and intensity of experienced emotions.
	Actions	O4	The tendency to choose novelty over the familiar.
	Ideas	O5	The degree of interest and curiosity in entertaining new thoughts and ideas.
	Values	O6	The willingness to re-evaluate norms and values.
Agreeableness	Trust	A1	The general level of wariness or suspicion in contact with other people.
	Straightforwardness	A2	Degree of sincerity vs shrewdness.
	Altruism	A3	Active concern for the well-being of others.
	Compliance	A4	Inhibiting vs expressing aggression towards others in conflict.
	Modesty	A5	Degree of humility vs arrogance.
	Tender-Mindedness	A6	Propensity to empathize with others.
Conscientiousness	Competence	C1	Belief in one’s own capacity to handle life’s many challenges.
	Order	C2	Degree of neatness and orderliness.
	Dutifulness	C3	How strongly ethical principles guide action.
	Achievement Striving	C4	Aspiration-level, the willingness to work towards goals.
	Self-Discipline	C5	The ability to follow through on tasks despite boredom.
	Deliberation	C6	How well one thinks things through before taking action.

### Procedure

Psychiatric assessments were conducted by trained and experienced clinicians through *Stepwise*, a standardized internet-based quality assurance protocol encompassing expert- and self-rated psychiatric instruments, including those listed above [35]. Due to differences in the assessment battery used at the day-patient unit at the start of the study period, involving the use of the EDI-2 instead of the EDE-Q, data on the EDE-Q was only available for 153 of 208 patients. Personality data was collected by posting the NEO PI-R self-report form and instructions to patients’ home addresses. The study was approved by The Regional Ethics Review Committee in Stockholm, reference number 2008/669-31.

## Results

Baseline characteristics are summarized in Table 2. Due to multiple comparisons alpha-level was set to p < .01for all analyses. To test whether patients with BN or EDNOS differed on personality, two-tailed t-tests were performed. Patients with BN or EDNOS did not differ significantly on any of the thirty facets, hence subsequently they were analysed jointly, as a single ED-group.

**Table 2:** **Baseline characteristics of patient sample**

**Characteristic**	**All patients (** ***N*** **= 153)**	**Bulimia nervosa (** ***N*** **= 99)**	**Eating disorder not otherwise specified (** ***N*** **= 54)**
Age	27.7 (7.9)	26.9 (7.6)	29.2 (8.2)
Body mass index	23.6 (4.7)	23.0 (3.6)	24.8 (6.0)
EDE-Q total score	3.95 (1.0)	3.95 (1.1)	3.96 (0.9)
CIA clinical impairment	28.1 (9.7)	28.2 (9.5)	27.9 (10.0)
CPRS			
Depression	9.4 (4.1)	8.8 (4.2)	10.3 (3.8)
Anxiety	8.4 (3.6)	8.1 (3.5)	9.0 (3.6)
Obsessive/compulsive	7.9 (3.6)	7.5 (3.7)	8.5 (3.5)

*Note*. CIA = Clinical Impairment Assessment, CPRS = Comprehensive Psychiatric Rating Scale, EDE-Q = Eating Disorder Examination Questionnaire.

### Personality profiles of patients and controls

Differences between ED patients and controls on personality facets were tested by two-tailed t-tests (Table 3). On the whole, ED patients differed significantly from controls on seventeen facets. The main pattern in patients compared to controls was that of greater Emotional Instability, decreased Gregariousness and Positive Emotions, less Openness to Actions and Ideas, and greater Openness in Values. They also reported reduced Trust, increased Modesty, lower Competence, and less Self-Discipline. Effect sizes of differences varied from small to very large, where large effect sizes were seen for facets related directly to emotional well-being, i.e. all six facets of Neuroticism and Positive Emotions.

**Table 3:** **Comparison of personality facets between patients (** ***N*** **= 208) and controls (** ***N*** **= 94)**

**Facet**	**Patients**	**Controls**	***t***	***p***	***d***
N1 Anxiety	21.8 (6.0)	16.9 (4.8)	−7.599	.000	1.02
N2 Angry hostility	17.6 (5.5)	12.7 (5.0)	−7.323	.000	0.85
N3 Depression	25.3 (5.5)	17.6 (4.8)	−11.748	.000	1.38
N4 Self-consciousness	19.7 (5.6)	14.7 (5.4)	−7.292	.000	0.85
N5 Impulsiveness	23.3 (4.8)	17.4 (4.7)	−9.998	.000	1.15
N6 Vulnerability	19.2 (5.4)	13.2 (5.4)	−8.970	.000	1.04
E1 Warmth	20.9 (5.0)	22.6 (4.4)	2.826	.005	0.32
E2 Gregariousness	17.9 (5.7)	20.8 (4.6)	4.680	.000	0.63
E3 Assertiveness	14.4 (5.5)	15.5 (4.2)	1.731	.085	-
E4 Activity	17.7 (5.3)	18.2 (4.2)	.763	.482	-
E5 Excitement-seeking	17.3 (5.2)	17.2 (4.4)	-.224	.823	-
E6 Positive emotions	18.5 (7.2)	23.2 (5.4)	5.720	.000	0.83
O1 Fantasy	19.0 (6.1)	19.2 (5.5)	.279	.780	-
O2 Aesthetics	16.9 (7.4)	18.1 (6.0)	1.481	.140	-
O3 Feelings	21.9 (4.9)	23.5 (4.2)	2.990	.003	0.41
O4 Actions	14.1 (5.5)	17.5 (5.0)	5.152	.000	0.59
O5 Ideas	16.5 (6.4)	19.0 (5.5)	3.350	.001	0.47
O6 Values	23.0 (3.8)	20.2 (3.0)	−6.241	.000	0.72
A1 Trust	17.1 (6.3)	19.5 (4.0)	3.857	.000	0.47
A2 Straightforwardness	19.1 (5.5)	20.3 (4.6)	1.843	.067	-
A3 Altruism	24.3 (4.5)	24.8 (3.7)	.984	.326	-
A4 Compliance	17.9 (5.4)	18.5 (3.7)	1.145	.253	-
A5 Modesty	21.9 (5.5)	19.7 (3.6)	−4.094	.000	0.50
A6 Tender-mindedness	22.7 (4.1)	22.6 (3.3)	-.221	.825	-
C1 Competence	17.8 (4.9)	21.3 (4.9)	5.836	.000	0.68
C2 Order	18.8 (5.2)	18.3 (4.6)	-.668	.504	-
C3 Dutifulness	21.5 (5.3)	22.3 (4.8)	1.230	.220	-
C4 Achievement striving	17.9 (5.2)	18.5 (4.2)	1.055	.293	-
C5 Self-discipline	15.3 (7.0)	19.5 (6.6)	4.985	.000	0.58
C6 Deliberation	15.9 (6.2)	17.5 (5.0)	2.331	.021	0.31

Data are shown as mean (SD). *d* = Cohen’s *d* effect size.

### Stepwise regression of personality on aspects of psychopathology

Univariate correlations between personality facets and indices of psychopathology were first calculated in order to identify independent variables for subsequent regression analysis. Correlations between facets and ED specific symptoms were generally weak, 10 facets showed a significant correlation with at least one symptom and/or with body mass index (BMI). Correlations between facets and indices of general psychopathology were stronger. Facets with significant univariate correlations with measures of psychopathology (*p* < .01) were then entered into stepwise regression analyses. In general, personality explained less of the variance in ED psychopathology than in general psychopathology (Table 4). Nevertheless, Depression and Warmth explained 12% of the variance in EDE-Q total score (*p* < .001). The ED symptom with the strongest relationship to personality was frequency of excessive exercise, where Achievement Striving, and Openness to Ideas (reversed) explained 10% (*p* < .001) of the variance. Activity had a shared variance of 4% with subjective binge eating (*p* < .01). No shared variance was found between personality and frequency of objective binge eating, loss of control over eating, or with purging. Personality facets explained 9-25% of variance in general psychopathology, as measured by CIA and CPRS. The facets Trust (reversed), Depression, Anxiety, and Vulnerability explained the largest proportion of variance. Positive Emotions (reversed), Order, and Gregariousness also helped to explain variance in general psychopathology, but were of less importance.

**Table 4:** **Summary of stepwise regression of symptom measures by patients’ NEO PI-R personality**

**Symptom measure**	**Personality facet**	**Adjusted R** ^**2**^	**F**	**β**	***p***
EDE total	N3 Depression	.081	10.7	.268	.001
	E1 Warmth	.115		-.199	.011
EDE objective binge eating	-	-	-	-	-
EDE loss of control	-	-	-	-	-
EDE subjective binge eating	E4 Activity	.040	7.2	.214	.008
EDE purging	-	-	-	-	-
EDE exercise	C4 Achievement Striving	.045	5.3	.235	.023
Body mass index	N1 Anxiety	.055	9.0	.254	.001
	E4 Activity	.096		-.216	.006
CIA clinical impairment	N6 Vulnerability	.065	8.1	.205	.015
	A1 Trust	.086		-.176	.036
CPRS depression	N3 Depression	.159	18.1	.348	.000
	E6 Positive Emotions	.220		-.267	.000
	C2 Order	.254		-.197	.006
CPRS anxiety	A1 Trust	.115	15.3	-.261	.001
	N3 Depression	.161		.242	.003
CPRS obsessive/compulsive	N1 Anxiety	.161	14.8	.264	.003
	E2 Gregariousness	.195		-.178	.018
	N6 Vulnerability	.218		201	.024

*Note*: Abbreviations explained in Table 2.

## Discussion

The present study aimed to explore how patients with non-anorexic ED differ from controls in personality facets of the Five-Factor Model and to examine the ability of personality facets to explain variance in ED and general psychopathology. ED patients differed from controls in the majority of facets, and facets from all five personality dimensions explained considerable amounts of variance in ED and general psychopathology. In general, the personality profiles of patients where characterised by the experience of pervasive negative affectivity and vulnerability, with little in the way of positive emotions such as joy, warmth and love. Patients reported a tendency to doubt their own capacity to deal with life’s challenges, were self-effacing, and believed other people are not to be trusted. They tended to avoid social gatherings and appeared to be less open to exploration, be it emotions, ideas or new activities. Curiously however, they rated themselves as undogmatic and less traditional. Finally, they reported a tendency to procrastinate and had trouble controlling desires, leading to rash action and regret.

The NEO PI-R profile of ED patients in this study shares commonalities with several other psychiatric disorders. Aside from Neuroticism and Positive Emotions, which do not discriminate well between diagnoses, a few interesting comparisons can be noted. Low Warmth and Gregariousness are distinctive of dysthymia, avoidant personality disorder (PD) and schizoid PD, while low Openness to Actions is common in generalized anxiety disorder (GAD), dysthymia, paranoid PD and avoidant PD. Low Trust is typical of several PDs and also typical of substance use disorder (SUD). Increased Modesty is common in dependent PD, and low Competence common in dysthymia, obsessive-compulsive disorder, SUD, depression, schizotypal, borderline and avoidant PD. Finally, low Self-Discipline can be seen in depression, dysthymia, GAD, SUD and in most PDs [24,36-38]. It is, therefore, not surprising that there is considerable overlap in personality facets in ED with other mental disorders, given that ED patients commonly suffer from comorbid axis-I [39] and II psychopathology [21], and certain maladaptive traits have been found to precede later ED development [13].

One possible way of understanding the marked and systematic differences in personality profiles between ED patients and controls is in terms of attachment theory. Insecure attachment is pervasive in ED patients [40], and Noftle and Shaver [41] have investigated how attachment relates to the FFM. Comparing their results with our findings, there are striking similarities in terms of the FFM. Noftle and Shaver also found similarities between the general profile of ED patients and people high in attachment anxiety and/or high in attachment avoidance. Their results suggest that high attachment anxiety is mainly positively related to Neuroticism and negatively related to Assertiveness, Competence, Dutifulness, Achievement Striving, Self-Discipline and Deliberation. Attachment avoidance, on the other hand, is inversely related mainly to Extraversion, Openness to Feelings, Trust, Competence and Self-Discipline. Taken together, the results of the present study and those of Noftle and Shaver suggest that ED treatment could benefit from targeting problematic personality traits and attachment difficulties, in addition to ED symptoms. By identifying and focusing on personality traits and attachment difficulties it may be possible for therapists enhance the treatment alliance, address important underlying problems, and ultimately improve outcome.

The current study is novel in exploring how FFM traits relate to psychiatric symptoms among ED patients. Personality explained 9-25% of the variance in general psychopathology and ED pathology. Three personality facets (Trust, Anxiety and Depression) shared the most variance with general psychopathology. In regards to severity of ED (as measured by total score on the EDE-Q), Depression and low Warmth explained 12% of the variance. We found no previous studies using the NEO PI-R with ED patients to compare our results with. However, a dissertation on bulimic features in female university students [42], found a shared variance of 23% between facets of Neuroticism and Excitement-Seeking and an ED severity measure (BULIT-R). Studies using the TCI have reported mixed results, both supporting [43], and refuting [44] a relationship between personality traits and symptom severity; interpretation of these studies must, however, consider the psychometric and theoretical weaknesses of the TCI mentioned previously. Regarding personality and specific ED symptoms, excessive exercise was related to high Achievement Striving and low Openness to Ideas. A recent case–control study on exercisers [45], confirms a relation between excessive exercise and high Achievement Striving. Clinically, patients who compensate frequently by exercising might hold perfectionistic/unrealistic strivings that need to be addressed. Patients low on Openness to Ideas, which can be expressed in terms of rigid thinking, may need help with devising alternative strategies to exercising for dealing with emotions.

Excitement-seeking, the need for adrenaline rushes and intense stimulation, has been implicated as a source of impulsive behaviours. Yet we did not detect elevated levels in patients. A meta-analysis on impulsivity and bulimic symptoms discovered that the primary motor behind impulsivity is not excitement-seeking but negative urgency; rash action in response to high levels of negative emotions. Excitement-seeking and deliberation shared second place [46]. Treatment should thus focus on devising a plan for handling challenging situations and to better cope with strong emotions when they arise. Excitement-seeking might not demand special attention, especially as it tends to recede with age (as in our ED sample, data not shown). Note of caution for this interpretation, as internal consistency for Excitement-Seeking was low.

This is a novel study in exploring the full FFM in outpatients with ED. Strengths of the study is sample size, the focus on the two largest ED populations, i.e. patients with BN or EDNOS, and contrasting them with a control sample. Comparing with age-matched controls, (instead of to FFM norms where mean age is 45 years) increases validity as age proved to be a major confounding factor for several personality facets. There are also several limitations. Being cross-sectional it was not possible to explore the interaction of traits and symptoms over time. The sample does not include patients of all ED diagnoses and the control sample was not screened for ED symptoms. No patients had AN, although some had a history of the disorder, which limits generalizability. Exploring the effect of comorbidity was outside the scope of the present study, although comorbidity with other psychiatric diagnoses is common among ED patients, and could naturally have an impact on both ED symptomatology and personality.

## Conclusions

The present study contributes new knowledge on the relationship between ED and the predominant personality trait model used today. Patients differ markedly from controls in terms of personality. Personality facets (especially trust, achievement striving, and facets of neuroticism) are important for understanding ED. By identifying and focusing on personality traits it may be possible for therapists enhance the treatment alliance, address underlying problems, and improve outcome. To further extend understanding of the interplay between personality traits and ED, future research should study their longitudinal relationship in response to treatment interventions.
